# The Value of Anti-Müllerian Hormone in the Prediction of Spontaneous Pregnancy: A Systematic Review and Meta-Analysis

**DOI:** 10.3389/fendo.2021.695157

**Published:** 2021-10-13

**Authors:** Chenxi Lin, Miaomiao Jing, Wenjun Zhu, Xiaoyu Tu, Qi Chen, Xiufang Wang, Youbing Zheng, Runju Zhang

**Affiliations:** ^1^ Key Laboratory of Reproductive Genetics (Ministry of Education), Department of Reproductive Endocrinology, Women’s Hospital, Zhejiang University School of Medicine, Hangzhou, China; ^2^ Medical Quality Management Section, Women’s Hospital, Zhejiang University School of Medicine, Hangzhou, China; ^3^ Department of Gynaecology and Obstetrics, Hangzhou Fuyang Women and Children Hospital, Hangzhou, China; ^4^ Department of Gynaecology, Zhejiang Xiaoshan Hospital, Hangzhou, China; ^5^ Department of Gynaecology, Shengzhou Maternal and Child Health Hospital, Shaoxing, China

**Keywords:** AMH, spontaneous pregnancy, meta-analysis, fertility, anti-Müllerian hormone

## Abstract

**Objective:**

To determine whether serum anti-Müllerian hormone (AMH) level is a predictor of clinical pregnancy in women trying to achieve a natural conception.

**Methods:**

The PubMed, Embase, and Cochrane Library databases were searched for articles published until August 2020. Studies that met the inclusion and exclusion criteria were included in the meta-analysis; no language limitations were imposed. Quality was appraised using the Quality Assessment of Diagnostic Accuracy Studies-2 checklist. Heterogeneity due to the threshold effect was identified; thus, we plotted a summary receiver operating characteristic curve and calculated its area under the summary receiver operating characteristic curve (AUC) and Cochran’s Q index to assess whether AMH level is a predictor of spontaneous pregnancy. Publication bias and sensitivity were also assessed.

**Results:**

Eleven studies (4,388 women) were ultimately included in this meta-analysis. The AUC and Cochran’s Q indices were 0.5932 and 0.5702, respectively. For women younger than 35 years, the AUC was 0.6355 and the Q index was 0.6025. For those older than 35 years, the AUC was 0.5536 and the Q index was 0.5403. Subgroup analyses by study type and population characteristics showed results similar to the overall outcome. No publication bias was identified, and the sensitivity analysis confirmed the robustness of the final result.

**Conclusions:**

Serum AMH levels have poor predictive value for natural pregnancy. The predictive value of AMH was poor in the younger and older subgroups. Our findings suggest that low serum AMH levels are not associated with reduced fertility.

**Introduction:**

This study investigated the predictive value of anti-Müllerian hormone (AMH) level for natural pregnancy. Other than age, few factors can predict the chances of natural fertility. AMH is an established biomarker of ovarian reserve that is widely used to predict oocyte yield in cases of *in vitro* fertilization (IVF) and menopause. In clinical practice, the applications of AMH are increasing. However, its predictive value for natural conception remains controversial. In this study, since AMH is closely related with ovarian reserve, we evaluated whether it has predictive value for natural pregnancy. Our findings will fine-tune the clinical application of AMH in pre-pregnancy counseling. The topic should be of wide interest to investigators in the reproductive endocrinology and gynecology fields.

**Systematic Review Registration:**

PROSPERO 2020 CRD42020216265, Available from: https://www.crd.york.ac.uk/prospero/display_record.php?ID=CRD42020216265.

## Introduction

Female fecundability declines with increasing age due to decreasing oocyte quality and quantity, also known as diminished ovarian reserve (DOR). Age is an independent predictor of ovarian reserve, and females in the late reproductive period usually have a lower chance of spontaneous pregnancy and worse pregnancy outcomes (1). Approximately 10% of women develop a latent ovarian function decline at a younger age, leading to undesirable reproductive outcomes. In such cases, DOR is not clearly identified, and the clinical manifestation shows a regular menstrual cycle but a lower response to ovarian stimulation than that of their peers. Moreover, an ancillary examination shows abnormal ovarian reserve test results. DOR treatment mainly focuses on oocyte quality, oocyte quantity, and fertility (2).

There are several biomarkers of ovarian reserve, such as anti-Müllerian hormone (AMH), follicle-stimulating hormone (FSH), and inhibit-B (3). AMH, a member of the transforming growth factor-β family that is secreted by the granulosa cells of preantral and antral follicles, reflects the follicle reserve and is considered one of the most established biomarkers of ovarian reserve. Compared to other biomarkers, AMH levels are more stable during the menstrual cycle (4). When ovarian reserve starts to decline, serum AMH level changes occur earlier than basal FSH level increases, and menstrual disorders develop. Thus, AMH level is thought to reflect decreased ovarian function early. In clinical practice, AMH level is widely used to predict menopause and reflect ovarian response in cases of assisted reproductive technology (ART) (5–7). However, existing studies have shown inconsistent viewpoints regarding its application in the prediction of natural conception. Several researchers have indicated that clinical doctors should consider AMH levels during fertility counseling, as low AMH levels appear to be a risk factor for a reduced natural pregnancy rate (8–13). However, other studies have reported the opposite result (14–21).

Although numerous case-control and cohort studies have been published to date, high-quality prospective cohort studies stratified by age that fully explore the predictive value of AMH in natural pregnancy are lacking.

Since previous studies presented inconsistent conclusions about AMH for predicting natural clinical pregnancy, this systematic review and meta-analysis aimed to identify whether serum AMH levels can predict natural clinical pregnancy in age-stratified women.

## Materials and Methods

### Search Strategy

This study was performed in accordance with PRISMA guidelines (22). This meta-analysis was registered in PROSPERO (ID: CRD42020216265). The PubMed, Embase, and Cochrane Library databases were searched for articles published until August 2020. The following keywords and subject terms were used: (Pregnancy OR Pregnancies OR Gestation OR Reproductive outcome OR Fertility OR Fecundability OR Conception) AND (AMH OR Anti-Muellerian Hormone OR Mullerian-Inhibiting Hormone OR Mullerian Regression Factor OR Mullerian Inhibiting Hormone OR Mullerian-Inhibitory Substance OR Anti-Mullerian Factor OR Mullerian-Inhibiting Factor OR Anti-Mullerian Hormone). The reference list of each identified primary study was also manually searched to ensure that all eligible studies were included in this meta-analysis. No language-related limitations were imposed.

### Eligibility Criteria and Exclusion Criteria

The inclusion criteria were as follows: (i) the study population included women of reproductive age and trying to get pregnant naturally for which the outcomes of clinical pregnancy within a year were recorded; (ii) serum AMH level was measured and study identify a low AMH cutoff value; (iii) women were recruited from the hospital or community; and (iv) sufficient information was available to construct the 2×2 contingency table—the true-positive, true-negative, false-positive, and false-negative test results at certain cutoff values. Considering that infertility is identified as the failure to achieve a successful pregnancy after 12 months or more of regular unprotected intercourse, studies comparing AMH levels of infertile and fertile women were included as well. If multiple publications reported the same or overlapping data, the most recent study with the largest population was included. If the same population was included in different studies with different selected AMH cutoff values, both studies were included in the meta-analysis. Studies were excluded if populations were restricted to women with diagnosed fallopian tube obstructive infertility, polycystic ovarian syndrome, or autoimmune disease. Reviews, conference abstracts, case reports, and comments were excluded from the study. Studies with insufficient or unavailable data were excluded from the analysis as well.

### Study Selection and Data Extraction

Two investigators (CL and MJ) independently screened all titles and abstracts. The full text of the preselected studies was read separately by the same two investigators to identify which met the inclusion criteria. Discrepancies were resolved by discussion with a third investigator (RZ).

The original data were collected separately by the two reviewers to avoid extraction errors. The characteristics of each study were extracted as follows: first author, year of publication, study type, population characteristics, patient ages, suggested AMH threshold (converted to ng/ml using the conversion formula ng/ml = 7.14 pmol/L), AMH assay, and number of true- and false-positive and -negative results. True-positive results were identified as failing to achieve natural pregnancy in a year with a low AMH serum level.

### Risk of Bias Assessment

The quality of the selected studies was assessed using RevMan 5.3 according to the QUADAS-2 checklist (23). The risk of bias of each study was divided into low, high, or unclear in terms of patient selection, index test, reference test, and flow and timing.

### Data Synthesis

The meta-analysis was performed using Meta-DiSc 1.4 software and STATA 12.0 software. The threshold effect, one of the most important causes of heterogeneity in diagnostic tests, was explored in Meta-DiSc 1.4 (24). The correlation between sensitivity and specificity was calculated to identify the threshold effect (25). A negative correlation (or positive correlation between sensitivities and 1-specificities), which results in a typical pattern of a “shoulder arm” plot in a summary receiver operating curve (SROC) space, suggests that different thresholds or cutoffs used in different studies cause the primary heterogeneity. If the threshold effect was present, the SROC curve was plotted, and its area under the curve (AUC) was calculated with the Cochran’s Q index. If no threshold effect is present, diagnostic odds ratio, sensitivity, specificity, and positive and negative likelihood ratios of AMH for predicting pregnancy were also generated. The Chi-square test was further used to explore heterogeneity other than the threshold effect, and the I-squared measure was used to quantify heterogeneity. The test level for the meta-analysis was set at α=0.05. Heterogeneity analyses were performed according to study type and population characteristics (e.g., risk factors for infertility). Deeks’ funnel and sensitivity analyses were also performed using STATA 12.0 software to analyze potential publication bias and the robustness of the results.

## Results

### Study Selection

**Figure 1:**
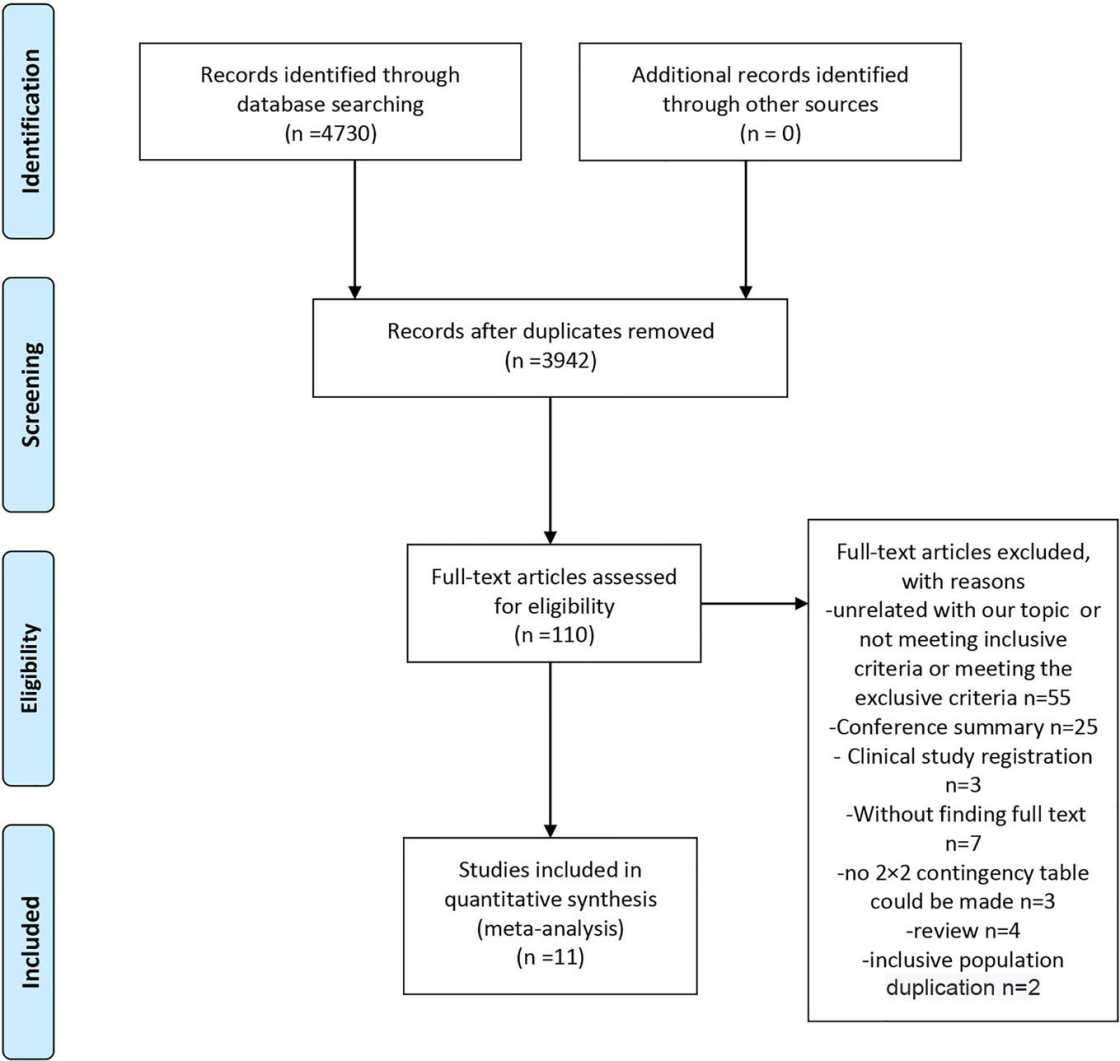
Flow of studies through the review.

A flowchart of the study selection process is shown in **Figure 1**. We searched the PubMed, Cochrane Library, and Embase databases and retrieved 4,730 pieces of literature. A total of 3,942 records remained after the removal of duplicates. After careful screening of the titles and abstracts, 110 studies remained and were subjected to the full-text review. In this process, 55 were excluded for the lack of relevance, not meeting the inclusion criteria, or meeting the exclusion criteria; 32 were excluded for article type (25 conference summaries, four reviews, and three clinical study registrations). Of the remaining studies, three did not include sufficient data to make a 2×2 contingency table, seven had unavailable full text, and two included a duplicate population. Finally, 11 studies were included in this meta-analysis (8, 11, 14–16, 26–31).

### Study Characteristics

The characteristics, inclusion criteria, and exclusion criteria of the 11 studies are listed in **Tables 1** and **2**. Of them, seven were prospective (8, 11, 14–16, 27, 31), two were case-control studies (15, 30), one was a retrospective cohort study (26), and one was a cross-sectional cohort study (28). Four other studies further included women over the age of 35 years (16, 28, 30, 31), while two focused on only women of younger reproductive age (≤35 years) (11, 14). Among the eligible studies, three included participants with one or more risk factors that may affect natural conception, such as ovarian surgery, endometriosis, or a history of infertility, while five studies did not and three studies compared the serum AMH levels in infertile women or those who achieved a pregnancy after more than 12 months of trying, to those with normal fertility.

**Table 1:** Characteristics of the studies included in the meta-analysis.

Study	Year	Study type	Population		AMH test		Outcome			
			Population characteristics	Age (y)	Threshold (ng/ml)	Assay	TP	FP	FN	TN
Korsholm	2018	Prospective cohort	–	No limitation	1.3	Elecsy	3	29	25	139
Casadei	2013	Prospective cohort	Unexplained infertility	No limitation	0.75^*^	IBC	22	5	47	9
Somigliana	2015	Prospective nested case-control study	–	No limitation	1.1^*^	GEN II ELISA	11	15	65	60
Murugappan	2019	Retrospective cohort	–	No limitation	1	NA	19	28	33	75
Casadei	2018	Prospective study	Ovary cyst	18–45	1.1	GENII ELISA	7	5	7	8
Steiner	2017	Prospective cohort	–	30–34	0.7	Ultrasensitive AMH ELISA	10	22	143	335
				35–44			21	31	85	90
Hagen	2012	Prospective cohort	–	<35	1.82^*^	GENI ELISA	16	20	60	90
Hvidman	2016	Case-control study: a prospective cohort study with a	–	<35	0.70^*^	GENI ELISA	8	6	235	232
		prospective cross-sectional study		35–40			10	12	129	100
Khan	2019	cross-sectional cohort	–	≤35	0.7	Elecsy	31	16	269	250
				36-39			25	18	98	104
Zhou	2019	Prospective cohort	Endometriosis	≤35	2	IBC	30	12	30	31
Zarek	2015	Prospective cohort	–	18–34	1	GEN II ELISA	22	54	346	640
				35–40			20	28	34	58

*Converted to ng/ml using the conversion formula ng/ml 7.14 pmol/L.

**Table 2:** Characteristics of the studies included in the meta-analysis.

Study	Inclusion criteria	Exclusion criteria		
**Korsholm**	(i) Women of reproductive age in a heterosexual relationship who had (ii) tried to conceive naturally or had an unplanned natural conception within 2 years after inclusion. All women included had (iii) a known duration of the pregnancy attempt, and (iv) AMH analyzed by the Elecsys^®^ method	Hormonal contraceptive use at inclusion		
**Casadei**	(1) Unexplained infertility, that is the lack of pregnancy after 1 year of unprotected sexual intercourse in women without apparent disorder of fertility; (2) normal or low ovarian reserve; (3) both ovaries present; (4) regular menstrual cycles	(1) PCOS according to the Rotterdam criteria (Rotterdam ESHRE/ASRM-Sponsored PCOS Consensus Workshop Group 2004); (2) congenital adrenal hyperplasia; (3) androgen secreting tumors; (4) Cushing syndrome; (5) male infertility; (6) tubal pathologies; (7) anovulation; (8) hyperprolactinemia; (9) hypothalamic amenorrhea; (10) previous ovarian surgery; (11) ovarian tumors; (12) anatomical abnormalities of the uterine cavity; (13) intraperitoneal adhesions; (14) endometriosis and other pelvic pathologies; (15) thyroid dysfunction and other endocrinological disorders such as diabetes mellitus; (16) recurrent pregnancy loss; (17) autoimmune diseases		
**Somigliana**	Inclusion criteria for both cases and controls were: (i) age >18 years, (ii) natural conception (women conceiving with the use of controlled ovarian hyperstimulation with or without assisted reproductive techniques were excluded), and (iii) regular menstrual cycles (24–35 days). Controls were the subsequently referred women matched to cases on the basis of age (6 months, ratio 1:1).	-		
**Murugappan**	Patients with a history of at least two prior pregnancy losses, defined as loss of pregnancy from conception through 20 weeks gestational age, were included.	-		
**Casadei**	Age between 18 and 45 years; ultrasound diagnosis of uni- or bilateral ovarian cysts; absence of malignancy criteria by ultrasound; and absence of endocrine disorders such as thyroid dysfunction, hyperprolactinemia, or Cushing syndrome	Histologic diagnosis of malignancy and perform bilateral ovariectomy; previous adnexal and uterine surgery or chemotherapy; and premature ovarian failure (POF)		
**Steiner**	Women between 30 and 44 years of age had been attempting to conceive for 3 months or less and were cohabitating with a male partner	Fertility problems (history of sterilization, diagnosis of polycystic ovarian syndrome, previous or current use of fertility treatments, known tubal blockage, surgically diagnosed endometriosis) or a partner with a history of infertility. Women who were currently breastfeeding or had used injectable hormonal contraception in the preceding year were also excluded		
**Zarek**	Women in this cohort were attempting pregnancy; were aged 18–40 years, with regular menstrual cycles of 21–42 days in length; and had a history of one to two prior pregnancy losses	history of infertility, pelvic inflammatory disease, tubal occlusion, endometriosis, anovulation, uterine abnormality, or polycystic ovarian syndrome		
**Hagen**	20–35 years old, lived with a partner, and had no children. Couples with no previous reproductive experience who intended to discontinue contraception to become pregnant were eligible for enrolment	-		
**Hvidman**	Study group: infertile patients referred for fertility treatment at The Fertility Clinic, Rigshospitalet, at Copenhagen University Hospital from September 2011 to October 2013. From September 2011, the Fertility Clinic offered newly referred infertile patients an assessment of ovarian and endocrine parameters prior to the first treatment cycle. Patients identified as eligible for the present study were examined on Cycle Days (CD) 2–5 and interviewed to obtain relevant background information. Control group: non-users of hormonal contraception with no history of infertility recruited in a prospective cross-sectional study conducted at the Fertility Clinic, Rigshospitalet, from August 2008 to February 2010.		Study group: The following patients were considered non-eligible: (i) patients referred for preimplantation genetic diagnosis, (ii) patients referred due to HIV or contagious hepatitis B or C infection, and (iii) single and homosexual women, as they were per se not considered infertile. Furthermore, patients referred directly for oocyte donation (OD) from other fertility centers were not examined on CD 2–5 and thus not included as they had already been diagnosed with a diminished ovarian reserve and most had started hormone replacement therapy or treatment with estradiol to prepare for the OD. Control group: polycystic ovary syndrome (PCOS) defined as oligo- or amenorrhea in addition to AFC ≥12 and/or an ovarian volume.10 ml3 in at least one ovary in accordance with the Rotterdam Criteria
**Khan**	i) no history of gynecological and abdominal surgery, ii) having the normal sonographic texture of ovaries, and iii) with no signs of hyperandrogenemia.		i) Those having any communicable disease or metabolic syndrome, ii) patients referred for pre-implantation genetic testing, iii) patients with polycystic ovarian syndrome (PCOS) and oligo-amenorrhea, iv) patients using any contraceptives, v) those having iatrogenic and autoimmune conditions, vi) obese infertile patients over the age of 40.
**Zhou**	Patients with an age of 20 to 35 years and a plan to conceive after surgery		Any suspicious findings of malignant disease, recurrent endometriosis, and hormone therapy within 3 months before surgery

### Synthesis of Results

**Figure 2:**
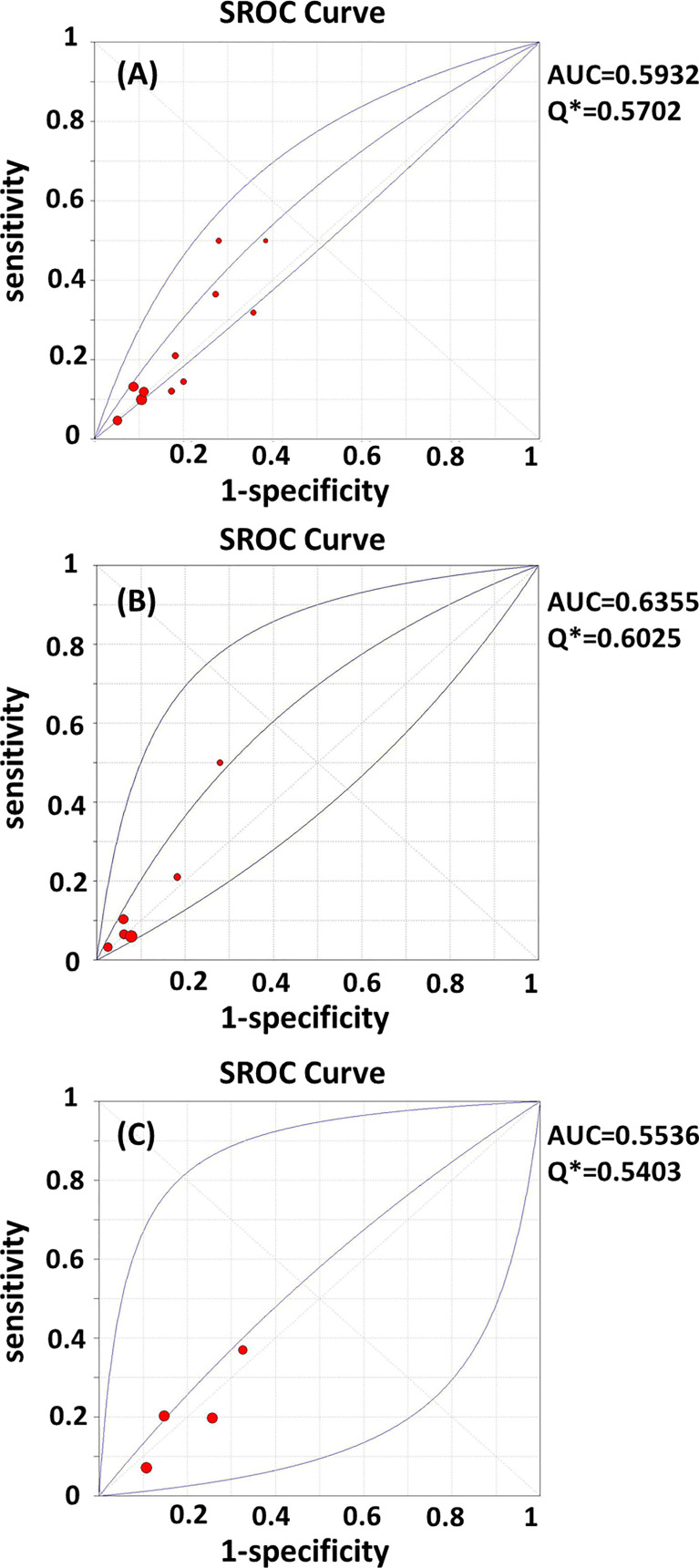
**(A–C)** SROC curves of AMH in the prediction of spontaneous clinical in **(A)** all women; **(B)** women younger than 35 years; **(C)** women elder than 35 years. AUC, area under the summary receiver operating characteristic curve; Q*,Cochran’s Q index.

**Figure 3:**
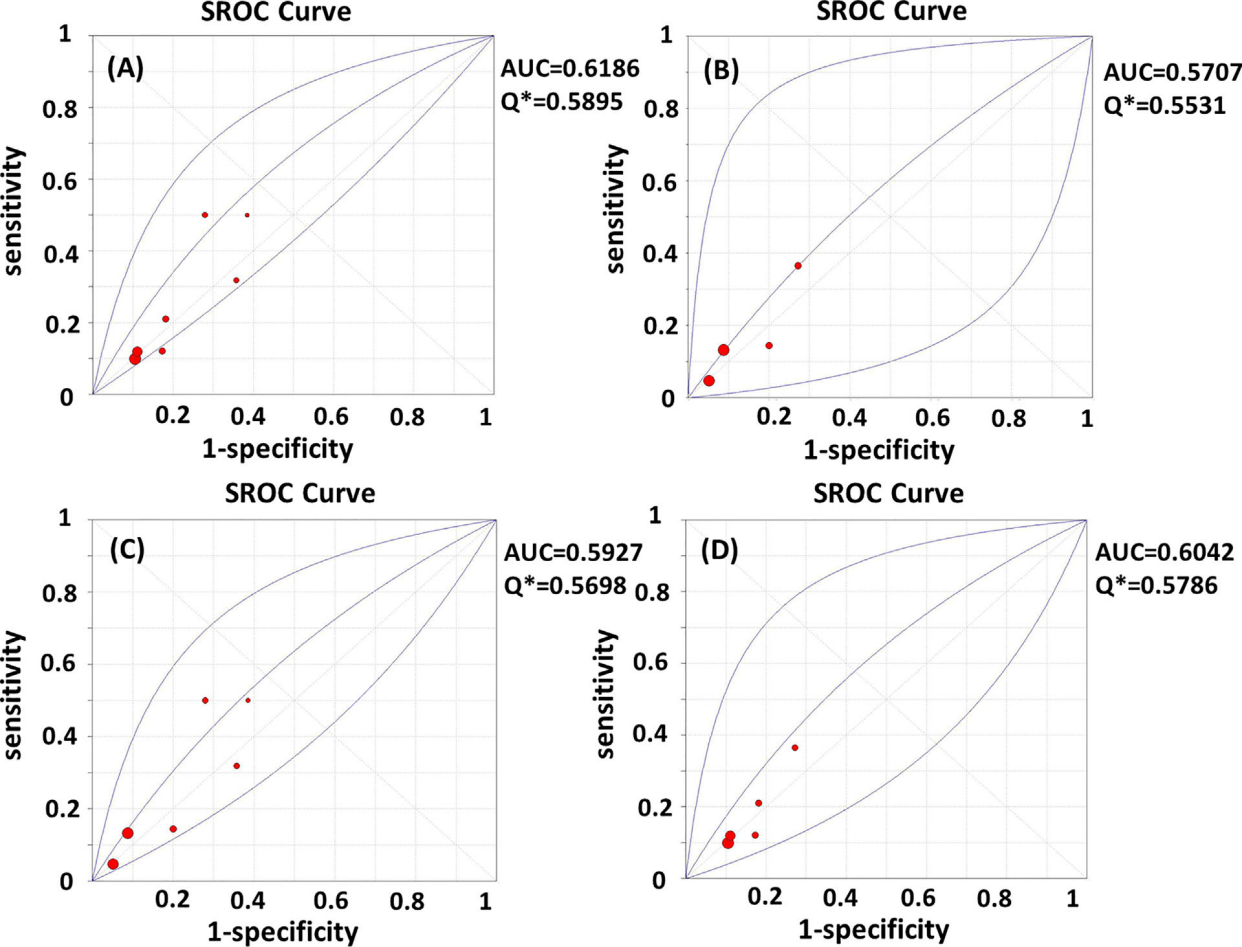
**(A–D)** SROC curves of AMH in the prediction of spontaneous clinical in **(A)** prospective subgroup; **(B)** non-prospective subgroup; **(C)** subgroup with risk factors for infertility; **(D)** subgroup without risk factors for infertility. AUC, area under the summary receiver operating characteristic curve; Q*, Cochran’s Q index.

Test heterogeneity presented a threshold effect in these 11 studies (n=4,388, Spearman correlation coefficient=0.902, p<0.01). Thus, we plotted an SROC curve and calculated its AUC and Q index, which were 0.5932 and 0.5702, respectively (**Figure 2A**). We further stratified this analysis into age subgroups. Six studies (n=2,908) were included in the young group, with an AUC of 0.6355 and a Q index of 0.6025 (**Figure 2B**). Four studies (n=863) were included in the elderly group, with an AUC of 0.5536 and Q index of 0.5403 (**Figure 2C**). When the studies were categorized by type, seven (n=2,539) were included in the prospective group, while four studies were included in another group (retrospective cohort, case-control, and cross-sectional studies; n=1,849). The AUC was 0.6186 *vs.* 0.5707, while the Q index was 0.5895 *vs.* 0.5531, respectively (**Figures 3A, B**). Among the eligible studies, six included participants with one or more risk factors that may affect natural conception, such as ovarian surgery, endometriosis, and history of infertility (n=1,907), and the AUC was 0.5927 and the Q index was 0.5698 (**Figure 3C**). Five studies included participants without known risk factors that may affect natural conception (n=2,481), with an AUC of 0.6042 and a Q index of 0.5786 (**Figure 3D**).

### Risk of Bias of Included Studies

**Figure 4:**
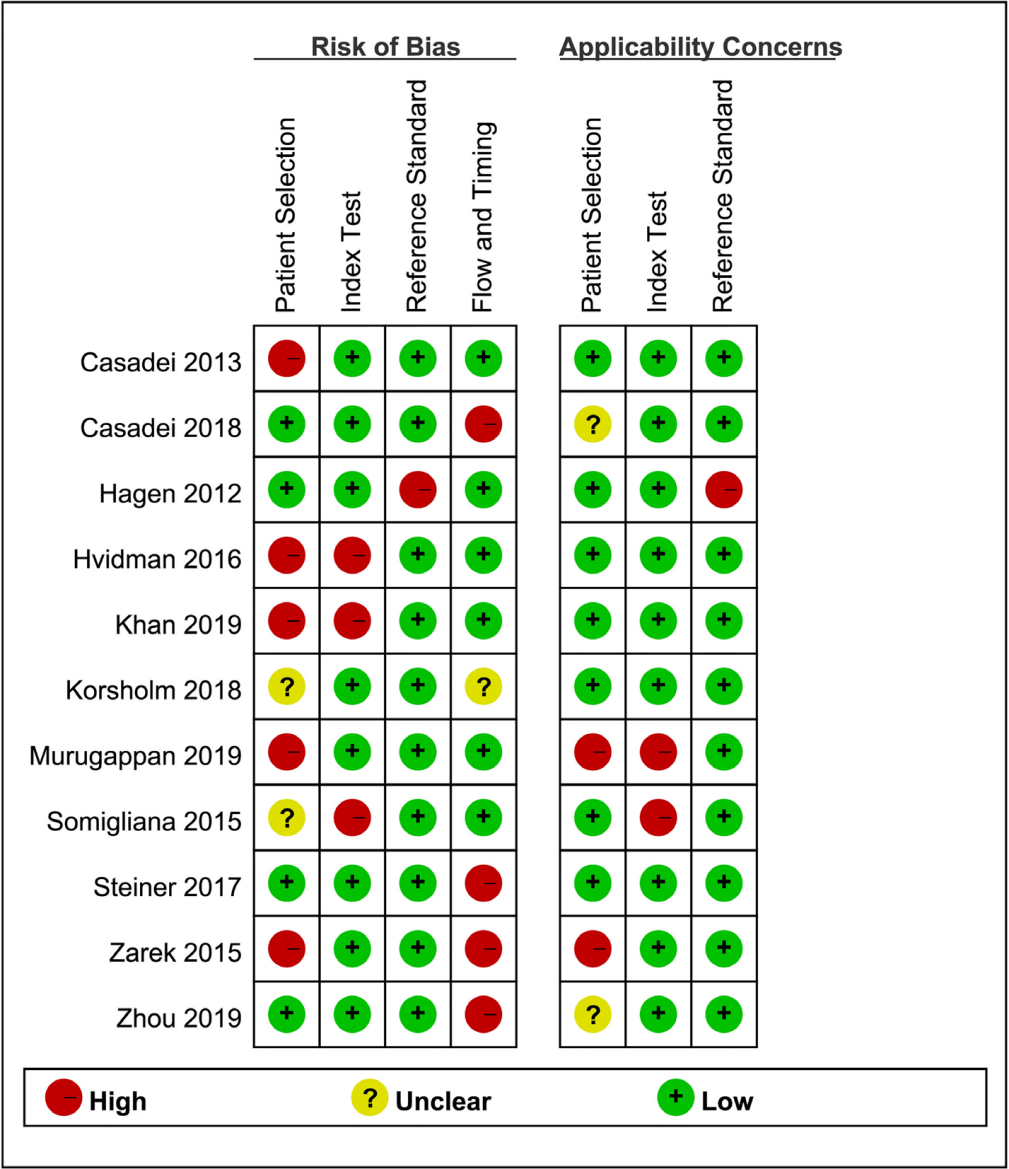
Risk of bias and clinical applicability of the included studies.

**Figure 5:**
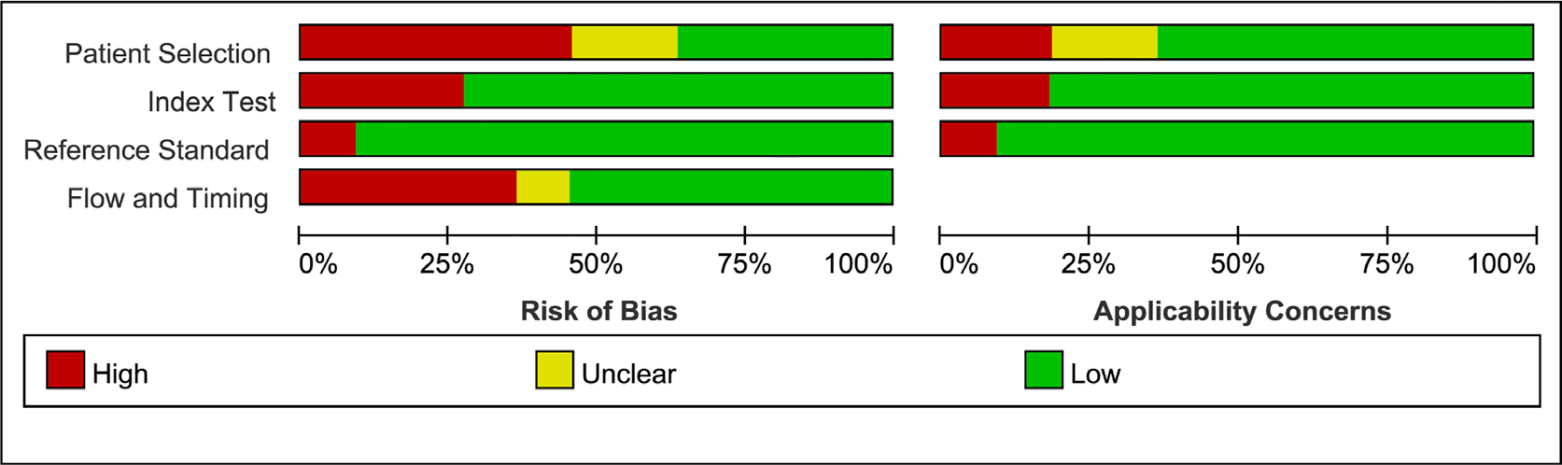
Graphical display for QUADAS-2 results.

**Figure 6:**
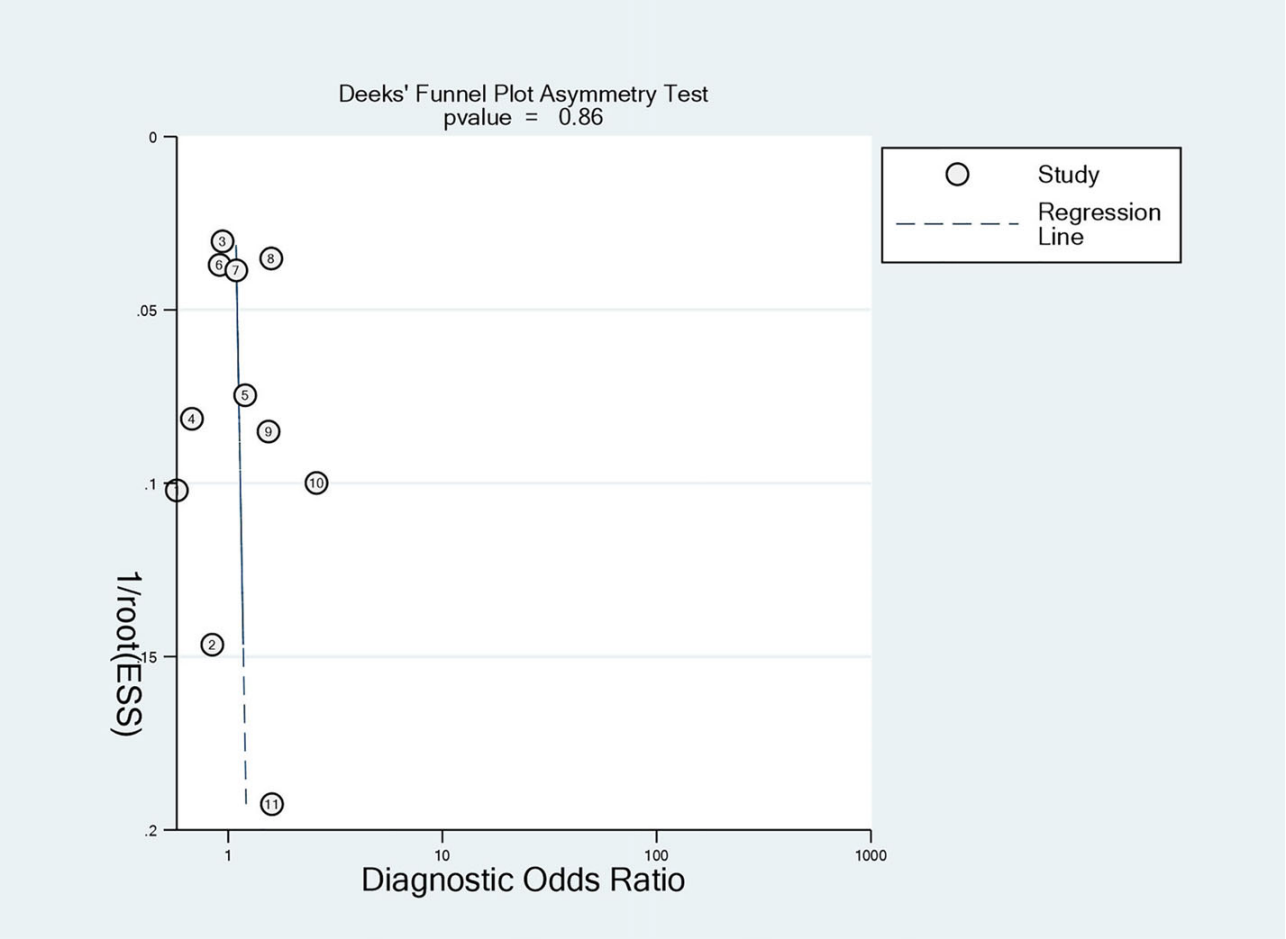
Deeks funnel for publication of included studies.

**Figure 7:**
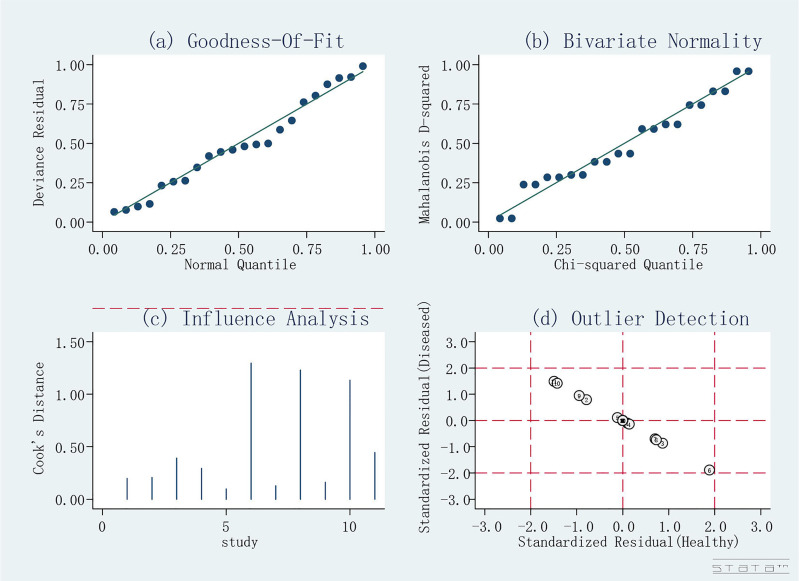
Sensitivity analysis.

The quality assessment of the included studies is shown in **Figures 4** and **5**. Deeks’ funnel plot did not suggest publication bias (p>0.05, **Figure 6**). The sensitivity analysis confirmed the robustness of the calculated results (**Figure 7**). Moreover, a subgroup analysis of study type and population characteristics revealed the same results.

## Discussion

Our meta-analysis indicated a weak predictive value of AMH for spontaneous pregnancy. The heterogeneity analysis confirmed the robustness of the calculated results. Calculations performed after the age stratification did not show an increased predictive ability.

AMH directly reflects the original follicular pool, and its secretion is unaffected by FSH, possessing the advantages of sensitivity and reliability. AMH has gradually replaced basic FSH as the most reliable ovarian reserve biomarker (3). Despite being one of the most widely used ovarian reserve tests in the clinical setting, a uniformly accepted low AMH cutoff is still lacking (3, 32), and the current primary reference is the Bologna Standard of AMH < 0.5–1.1 ng/ml.

In this meta-analysis, the included studies chose different cutoff AMH values detected by different AMH assays of 0.75–2 ng/ml. As a result, a negative correlation between sensitivity and specificity (known as the threshold effect) was presented. To prevent the benefits of the experimentation from being exaggerated, we plotted the SROC curve. The predictive value was limited when the AUC was 0.5–0.7, better when it was 0.7–0.9, and best when it was >0.9. The overall AUC was 0.5932 and the Q index was 0.5702, suggesting a weak predictive ability.

A woman’s serum AMH concentration usually peaks at around 20–25 years of age and gradually decreases with age to undetectable levels (33). The age-related ovarian function decline is generally accompanied by menstrual cycle disorders, ovulation disorders, and oocyte quality reduction, which account for approximately 10% of female infertility cases (34). In addition, decreased estrogen levels also adversely impact endometrial receptivity, the pelvic microenvironment, and other factors, which also leads to a decline in female fertility (34). To fully explore the predictive value of AMH for natural fertility, participants were stratified into older or younger than 35 years of age subgroups. Four studies divided the included participation by age (16, 28, 30, 31), while two studies included only women under 35 years of age (11, 14). An increased AUC and Q index were found in women younger than 35 years of age (0.6355 and 0.6025, respectively). Considering that the AUC was still lower than 0.7, AMH has weak predictive value for spontaneous pregnancy in young women. In women older than 35 years of age, the AUC was 0.5536 and the Q index was 0.5403, indicating poor predictive value.

Our age-stratified results indicated that low serum AMH levels do not necessarily represent decreased natural fertility in younger or older women. Statistics on age-specific AMH levels in Korean women with regular menstrual cycles show that women older than 35 years of age have an AMH level lower than 1.5 ng/ml, a finding that is consistent with the cutoff values that most included studies chose (0.75–2 ng/ml) (35). In younger women, an early decrease in AMH levels suggests an abnormally declined ovarian reserve, which might lead to decreased fertility. However, our meta-analysis showed that the predictive value of AMH for spontaneous pregnancy in this group was limited. AMH levels may be more closely associated with follicle quantity than oocyte quality in young women, which is inferred in some IVF-related studies as well (36–38).

Compared to ovarian reserve, regular ovulation and oocyte quality may hold greater significance in spontaneous pregnancy. Our meta-analysis did not further stratify AMH levels into low *versus* extremely low AMH because of the limited number of original studies. Some researchers believe that active treatment could be considered for young women with extremely low AMH levels, except for those with low AMH levels but no infertility factors (39, 40). Unfortunately, few original studies further divided AMH levels into low and extremely low subgroups; thus, we failed to determine whether there is an improved predictive value. In women of later reproductive age, a significant reduction in ovarian reserve is part of the biological progress due to the accelerated depletion of the follicular pool. Thus, the serum AMH concentration varied from low to undetectable. In this group, a relatively higher AMH concentration might enable a slower follicular failure rate and better conception ability.

A previous meta-analysis synthesized the effect of AMH on implantation, clinical pregnancy, and live birth in IVF, and its results demonstrated that the predictive effect was weak overall, although better but still low in women with DOR. Most studies defined DOR as age >35 years (41, 42). Contrary to the expected outcome, this meta-analysis does not determine a reliable predictive value in women aged >35 years. A decreased AMH level does not indicate decrease natural fertility either. A possible reason for this finding might be that only the oocyte quantity experiences a slower depletion.

A committee opinion on ovarian reserve tests indicated that a suitable crowd should be fully considered. As a screening test, AMH would be more applicable to the general IVF population as well as women at a high risk of DOR than women at a lower risk of DOR (3). However, AMH is more universally applied in clinical practice. Therefore, a confirmed quantitative or qualitative relationship between AMH and natural pregnancy is necessary for clinicians to provide individualized fertility guidance. The findings of our meta-analysis might be complementary to previous opinions about natural pregnancy. Meanwhile, it is important to avoid unnecessary fertility anxiety among reproductive-aged females, especially young nulliparas with decreased serum AMH levels.

To our knowledge, this meta-analysis is the first to examine the effects of AMH for natural pregnancy prediction. Furthermore, we also analyzed the effects in young *versus* old subpopulations. However, this study has several limitations. First, most primary studies analyzed AMH levels as predictors of reproductive outcomes using a certain cutoff value. However, in clinical practice, the use of only one cutoff value probably does not reflect the biological situation. We considered the factor of age; however, in each age range, further stratification of AMH values may show increasing predictive value. Unfortunately, studies dichotomized the data using a certain cutoff value, resulting in a non-differential classification error. Second, not all studies were performed in an age-stratified manner. The limited number of subgroup studies might affect the accuracy of the SROC curve. In addition, the included studies were mainly from western countries, which might restrict the application of their findings to other races. Additionally, different AMH assays were performed. Incomparable values and measurement deviations may influence the conclusions. Considering that our meta-analysis only assessed the predictive value of AMH for natural pregnancy, its predictive value for other important reproductive outcomes, such as live birth, requires further exploration.

In conclusion, the findings of existing studies vary regarding whether AMH levels can predict natural pregnancy, and our meta-analysis suggested weak predictive value of serum AMH level for natural clinical pregnancy. A decreased AMH level does not represent decreased natural fertility in young or old females. Thus, caution should be exercised regarding the appropriate application of AMH measurements, especially in pre-conception counseling, to avoid over-interpreted and unnecessary fertility anxiety.

## Author Contributions

CL performed the literature screening, the data extraction, the analyses, and the drafting of the manuscript. MJ performed the literature screening, data extraction. CL and MJ have contributed equally to this work. WZ reviewed the protocol. XT and QC drafted the protocol. XW and YZ extracted the baseline characteristic. RZ contributed to the literature inclusion and revised the manuscript for important intellectual content. All authors contributed to the article and approved the submitted version.

## Funding

Zhejiang University Education Foundation Global Partnership Fund, Fundamental Research Funds for the Central Universities, Key Research and Development Program of Zhejiang Province (No. 2021C03098).

## Conflict of Interest

The authors declare that the research was conducted in the absence of any commercial or financial relationships that could be construed as a potential conflict of interest.

## Publisher’s Note

All claims expressed in this article are solely those of the authors and do not necessarily represent those of their affiliated organizations, or those of the publisher, the editors and the reviewers. Any product that may be evaluated in this article, or claim that may be made by its manufacturer, is not guaranteed or endorsed by the publisher.
